# Machine Learning-Driven Discovery and Evaluation of Antimicrobial Peptides from *Crassostrea gigas* Mucus Proteome

**DOI:** 10.3390/md22090385

**Published:** 2024-08-26

**Authors:** Jingchen Song, Kelin Liu, Xiaoyang Jin, Ke Huang, Shiwei Fu, Wenjie Yi, Yijie Cai, Ziniu Yu, Fan Mao, Yang Zhang

**Affiliations:** 1 CAS Key Laboratory of Tropical Marine Bio-Resources and Ecology and Guangdong Provincial Key Laboratory of Applied Marine Biology, South China Sea Institute of Oceanology, Chinese Academy of Sciences, Guangzhou 510301, China; songjingchen21@mails.ucas.ac.cn (J.S.); jinxiaoyang23@mails.ucas.ac.cn (X.J.); huangke23@mails.ucas.ac.cn (K.H.); fushiwei21@mails.ucas.ac.cn (S.F.); yiwenjie20@mails.ucas.ac.cn (W.Y.); caiyijie19980802@126.com (Y.C.); carlzyu@scsio.ac.cn (Z.Y.); 2 College of Marine Life Sciences, Ocean University of China, Qingdao 266100, China; liukelin@stu.ouc.edu.cn; 3 University of Chinese Academy of Sciences, Beijing 100049, China

**Keywords:** machine learning, bioactive prediction, marine antimicrobial peptides, oyster mucus proteome

## Abstract

Marine antimicrobial peptides (AMPs) represent a promising source for combating infections, especially against antibiotic-resistant pathogens and traditionally challenging infections. However, traditional drug discovery methods face challenges such as time-consuming processes and high costs. Therefore, leveraging machine learning techniques to expedite the discovery of marine AMPs holds significant promise. Our study applies machine learning to develop marine AMPs, focusing on *Crassostrea gigas* mucus rich in antimicrobial components. We conducted proteome sequencing of *C. gigas* mucous proteins, used the iAMPCN model for peptide activity prediction, and evaluated the antimicrobial, hemolytic, and cytotoxic capabilities of six peptides. Proteomic analysis identified 4490 proteins, yielding about 43,000 peptides (8–50 amino acids). Peptide ranking based on length, hydrophobicity, and charge assessed antimicrobial potential, predicting 23 biological activities. Six peptides, distinguished by their high relative scores and promising biological activities, were chosen for bactericidal assay. Peptides P1 to P4 showed antimicrobial activity against *E. coli*, with P2 and P4 being particularly effective. All peptides inhibited *S. aureus* growth. P2 and P4 also exhibited significant anti-*V. parahaemolyticus* effects, while P1 and P3 were non-cytotoxic to HEK293T cells at detectable concentrations. Minimal hemolytic activity was observed for all peptides even at high concentrations. This study highlights the potent antimicrobial properties of naturally occurring oyster mucus peptides, emphasizing their low cytotoxicity and lack of hemolytic effects. Machine learning accurately predicted biological activity, showcasing its potential in peptide drug discovery.

## 1. Introduction

The escalating threat of antimicrobial resistance presents a significant public health challenge in the 21st century [1]. Addressing this pressing issue, molecules of natural origin, such as antimicrobial peptides (AMPs), have emerged as promising candidates for antimicrobial agents. AMPs play pivotal roles in the innate immune systems of diverse organisms, exhibiting potent, rapid, and broad-spectrum antimicrobial activity against bacteria, viruses, fungi, and parasites [2,3,4,5]. Notable examples include gramicidin, sourced from the *Brevibacillus* soil bacterium [6]; purothionin from wheat (*Triticum aestivum* L.) endosperm [7]; bombinin from frogs [8]; and cecropins from insects [9]. The current Antimicrobial Peptide Database (https://aps.unmc.edu/AP/, accessed on 12 June 2024) contains records of over 3900 such peptides, underscoring their substantial potential for therapeutic applications targeting microbial infections, cancer, inflammatory conditions, and metabolic disorders [10].

Ocean creatures represent more than half of the biodiversity worldwide. One of the most significant advantages of marine antimicrobial peptides (AMPs) is their low cytotoxicity and lack of hemolytic properties, making them highly attractive for marine drug development [11]. The field of marine drug research dates back to the 1950s with the discovery of the antiviral and anticancer drugs Ara-A and Ara-C from the sponge *Cryptotethya crypta* [12]. A notable milestone in this field is Et-743 (Trabectedin), derived from the ascidian *Ecteinascidia turbinata*, which is celebrated for its complex molecular structure and favorable therapeutic efficacy [13]. AMPs found in fish mucus, including families such as hepcidins, β-defensins, histone-derived peptides, cathelicidins, and piscidins, have shown potential as immunostimulants in aquaculture [14,15]. In marine arthropods, shrimp anti-lipopolysaccharide factor (SALF) and crab tachyplesin exhibit both antimicrobial and anticancer activities [16,17]. Among molluscs, mussels have been identified as rich source of antimicrobial compounds, yielding over 10 AMPs in the past decade. Noteworthy examples include peptides from *Perna canaliculus*, which possess antioxidant and angiotensin-converting enzyme (ACE) inhibitory properties [18].

The pursuit of bioactive peptides has also garnered considerable scientific interest in oysters [19,20]. Mucus analysis of *Crassostrea gigas* and *Ostrea edulis* revealed the presence of proteins implicated in microbe neutralization or detoxification processes, such as bactericidal/permeability-increasing protein [21,22]. For instance, in *C. gigas* mucus, DM9CP acts as a pattern recognition receptor, binding to a range of microbial entities [23]. Defensin, synthesized in *C. virginica* gill epithelial cells, was found in moderate levels in mucus, and demonstrates potent antibacterial activity against both Gram-positive and Gram-negative bacteria including *Staphylococcus aureus* and *Vibrio parahemolyticus* [24,25,26]. The proteomic analysis of the mucous proteins in *C. virginica* identified 1514 proteins, of which over 200 were categorically defined with specific functions such as immune activation and cell signaling. However, the majority of these proteins remain functionally uncharacterized. Mucous proteins, as reservoirs for antimicrobial peptides, yield proteolytically derived peptides with antimicrobial properties. However, the variability in physiological conditions leads to differences in these peptide products, making it challenging to precisely identify the specific peptides generated through enzymatic degradation or proteolysis. Recently, a study in directly predicting active peptides employed bioinformatics algorithms to unveil 2603 polypeptides with antimicrobial properties within the human proteome, originating predominantly from proteins encompassing diverse biological functions or peptide hormones with undisclosed functionalities [27], which provides us a promising train of thought for further mining active peptides from oyster mucous proteome.

Herein, we identified over 4000 proteins from the mucus of oysters. To investigate unknown antimicrobial components in the mucus, we extracted a dataset comprising more than 40,000 peptide sequences ranging from 8 to 50 amino acids in length from the mucous protein pool. These peptides were preliminarily ranked based on hydrophobicity, charge, and other properties of amino acids for their antimicrobial potential. Subsequently, following the approach of Jing et al. [28], we employed machine learning techniques to study and predict the antimicrobial activity, hemolytic activity, toxicity, and other characteristics of these peptide sets. We further screened polypeptides with broad-spectrum antibacterial activity, low toxicity, and low hemolytic activity and conducted in vitro functional analysis of six polypeptide sequences. The results highlight the potential of machine learning in mining antimicrobial peptides from shellfish and lay a solid theoretical foundation for screening marine drug candidates.

## 2. Results

### 2.1. Mucus Proteome Functional Enrichment Analysis

**Figure 1:**
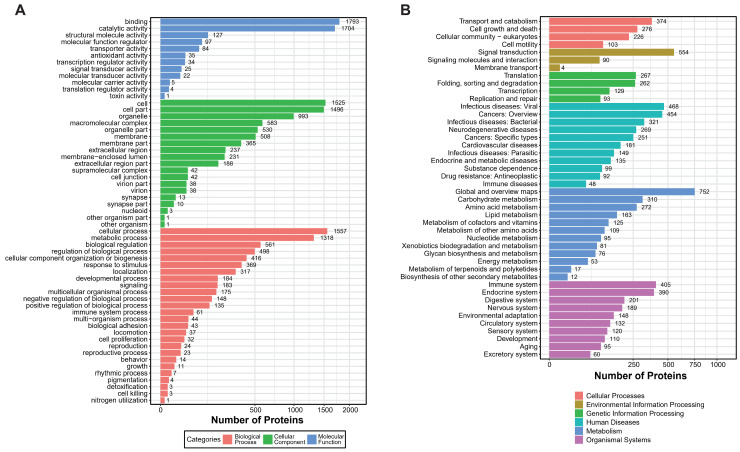
GO and KEGG enrichment analysis of *Crassostrea gigas* mucous proteins. (**A**) GO enrichment analysis shows three main categories of significant enrichment. Three colors illustrate the enrichment outcomes: blue, molecular function; green, cellular component; red, biological process. Categories are presented in descending order based on the number of enriched protein counts. (**B**) KEGG enrichment analysis. The six colors depict different categories: red, cellular processes; yellow, environmental information processing; green, genetic information processing; light blue, human diseases; dark blue, metabolism; purple, organismal systems. Pathways are listed in descending order based on the number of enriched peptide counts.

Using mass spectrometry analysis of mucus samples from healthy oysters, we identified 18,642 peptide segments and 4490 proteins, applying a stringent filtering criterion of FDR (False Discovery Rate) ≤ 1% (Table 1). Based on this proteomic dataset, we conducted GO (Gene Ontology) and KEGG (Kyoto Encyclopedia of Genes and Genomes) enrichment analyses to gain deeper insights into the complex biological processes and signaling pathways within the mucus. The GO analysis revealed that over 1700 proteins exhibit binding and catalytic activities, with fewer proteins involved in the regulation of protein translation and toxicity. Additionally, approximately 1318 proteins participate in metabolic processes, and around 1500 proteins function within specific cellular structures or tissues, including about 500 proteins acting on cell membranes and 200 proteins performing extracellular functions (Figure 1A). In the KEGG enrichment analysis, proteins were significantly enriched in categories related to the immune system and signal transduction, highlighting their crucial roles in mucosal immunity (Figure 1B).

### 2.2. The Hydrophobicity Scales of Peptides and the Correlation of Length and Relative Scores

**Figure 2:**
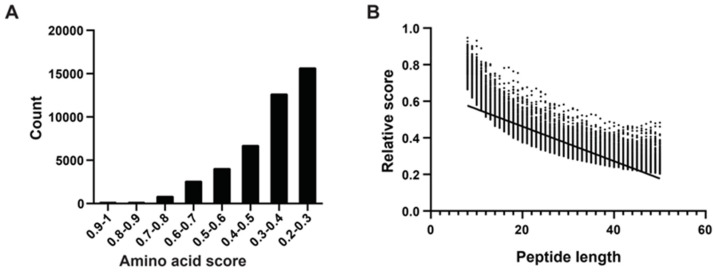
Amino acid physical and chemical properties score system, polypeptide fraction, and length correlation analysis. (**A**) According to Katia’s method, higher amino acid scores correspond to increased hydrophobicity, indicating stronger membrane affinity. The x-axis represents eight intervals of amino acid scores, and the y-axis represents the count of mucous peptides. (**B**) The relative score calculation formula incorporates the peptide’s net charge (C) and hydrophobic score (H), where higher positive charges and increased hydrophobicity contribute to a higher RS, indicating stronger membrane interaction capability. The x-axis represents peptide length (8–50 AAs) and the dots represent relative scores. The trend line highlights the trend of decreasing RS with increasing peptide length, emphasizing the impact of peptide size on its scoring metrics.

Following the method of Katia et al. [29], we determined amino acid scores using an algorithm that considered sequence length, net charge, average hydrophobicity, and other physicochemical properties. We randomly selected peptides ranging from eight to 50 amino acids in length from mucous proteins, obtaining 1000 peptides of each length category. In total, we generated 43,000 peptide sequences. We computed absolute and relative scores for all 43,000 peptides, categorizing them based on their relative scores, which predominantly fell within the range of 0.2 to 1.0. Notably, there were 10 peptides with scores between 0.9 and 1.0, all of which were eight to 10 amino acids long. The majority of peptides fell into the score ranges of 0.2–0.3 (15,718 peptides) and 0.3–0.4 (12,705 peptides), with longer lengths (Figure 2A). Subsequently, we conducted a correlation analysis between peptide length and relative scores, revealing a negative correlation where higher scores tended to correspond with shorter peptides (Figure 2B).

### 2.3. Performance of Peptide Sets Functional Activities According to iAMPCN

**Figure 3:**
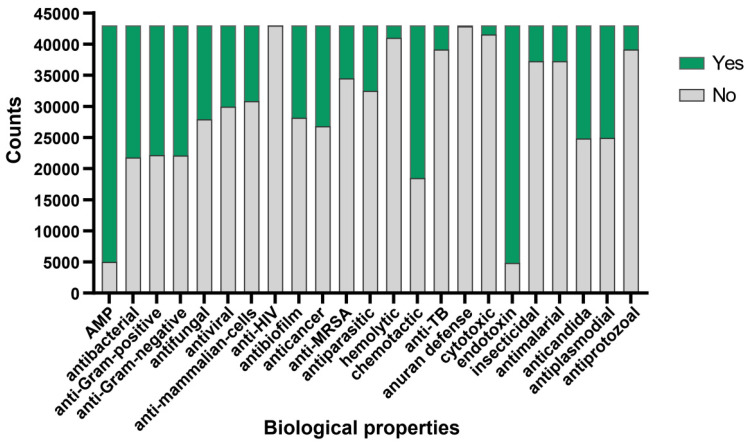
Bioactive properties of *Crassostrea gigas* mucous protein-derived peptides. The predicted functional activities encompassed AMP, antibacterial, anti-Gram-positive, anti-Gram-negative, antifungal, antiviral, anti-mammalian cells, anti-HIV, antibiofilm, anticancer, anti-MRSA, antiparasitic, hemolytic, chemotactic, anti-TB, anuran defense, cytotoxic, endotoxin, insecticidal, antimalarial, anticandida, antiplasmodial, and antiprotozoal properties. Green indicates the number of peptides possessing this functional characteristic, while grey indicates the number of peptides lacking the corresponding function.

To further validate the antimicrobial activity of the peptides evaluated by aforementioned algorithm, we adopted the deep learning approach described by Jing et al. [28]. Utilizing the iAMPCN learning framework, we evaluated the 43,000 peptides to predict the biological properties of bioactive peptides. The results showed that nearly 40,000 peptides exhibited endotoxin activity, with a similar number of peptides identified as antimicrobial peptides (AMPs). Approximately 1500 peptides exhibited hemolytic properties, with fewer than 1500 peptides showing cytotoxic effects. Furthermore, nearly 60% of peptides demonstrated chemotactic properties, while over 30% exhibited antibacterial, antifungal, antiviral, anticancer, and antibiofilm activities. A small subset of peptides displayed antiprotozoal, antimalarial, insecticidal, and anti-TB activities. Approximately 20,000 peptides showed potential in treating diseases caused by plasmodia or Candida infections. Regarding common characteristics, nearly all peptides were ineffective in the anuran defense mechanism and showed no activity against HIV (Figure 3). For detailed prediction results, please refer to Supplementary File S2.

### 2.4. Characterization of Candidate Peptide Sequences

**Figure 4:**
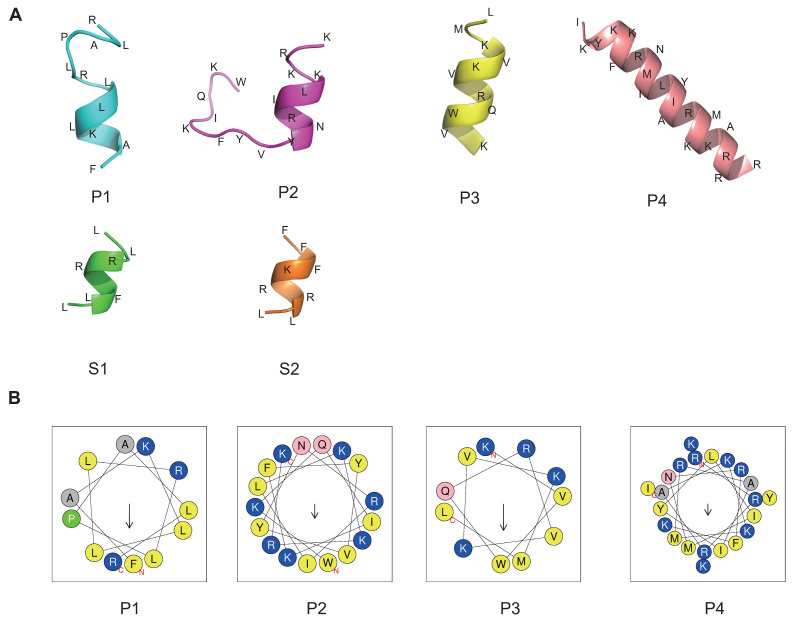
Three-dimensional structure and hydrophobic ring prediction of candidate peptides. (**A**) The peptide structure was predicted by the PEP-FOLD server. Amino acids are abbreviated. (**B**) Helical wheel projection. The structural motifs were shown as hydrophobic (yellow) and cationic (blue). Arrows inside each wheel represent the hydrophobic moment’s direction (μH).

To representatively analyze the reliability of our method and to screen the valuable peptides using optimal parameters, six peptides were arranged for synthesis and subsequent analysis, as shown in Table 2. The peptides with the top four highest relative scores and exhibiting broad-spectrum antibacterial activity, low toxicity, and minimal hemolytic activity were designated as P1 to P4. The two peptides achieving the highest RS were named S1 and S2, with scores of 0.947085 and 0.927233, respectively. To understand the antimicrobial mode of action, we performed three-dimensional structure predictions of the peptides, confirming the formation of α-helices in all six peptides, as illustrated in Figure 4A using the PEP-FOLD server. The projection of peptides P1, P2, P3, and P4 showed the distribution of hydrophobic and cationic residues. Positively charged amino acids, specifically Lysines and Arginines (depicted in blue), were prominently localized. Hydrophobic residues (shown in grey and yellow) were positioned on opposing sides of the wheel, creating a distinct hydrophobic moment (indicated by the arrow) within the peptide molecules (Figure 4B). However, HeliQuest was unable to predict the structure for shorter peptides like S1 and S2, which have eight or fewer amino acid residues.

### 2.5. Antimicrobial Effects of Six Polypeptides against Escherichia coli, Staphylococcus aureus, and Vibrio parahaemolyticus

**Figure 5:**
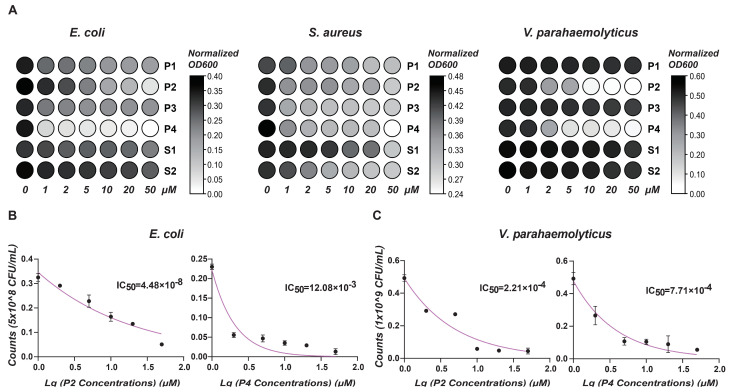
Antimicrobial activity analysis. (**A**) This heat map shows the effect of different antimicrobial peptide concentrations on OD value of bacterial growth. X-axis: peptide concentrations (μM). Y-axis: Normalized OD (600) values. Color intensity reflects OD changes: darker shades signify higher values; lighter shades, lower. (**B**) Antimicrobial activity of P2 and P4 at different concentrations (μM) against *E. coli*. (**C**) Antimicrobial activity of P2 and P4 at different concentrations (μM) against *V. parahaemolyticus*. Results show mean ± SD of bacterial counts (CFU/mL) and IC50 values.

To evaluate the antimicrobial activity of the six predictive peptides, we performed bacterial clearance assays against *Escherichia coli*, *Staphylococcus aureus*, and *Vibrio parahaemolyticus*. As depicted in Figure 5A, peptides P1 to P4 demonstrated significant effects against *E. coli*, with peptides S1 and S2 exhibiting slightly antimicrobial activity. At higher concentrations of P1 to P4, there was a substantial reduction in bacterial absorbance, particularly highlighting the pronounced antimicrobial effects of peptides P2 and P4. Concerning *S. aureus*, increasing peptide concentrations moderately slowed bacterial growth, with peptide P4 displaying the most substantial inhibitory effect among the tested peptides. For *V. parahaemolyticus*, peptides P2 and P4 exhibited marked, dose-dependent bactericidal effects, whereas the other four peptides showed minimal inhibitory effects on bacterial growth. Gradient killing assays further delineated the minimal inhibitory concentrations (MICs) of P2 and P4 (Figure 5B,C).

### 2.6. Evaluation of In Vitro Cytotoxicity and Hemolytic Activity

**Figure 6:**
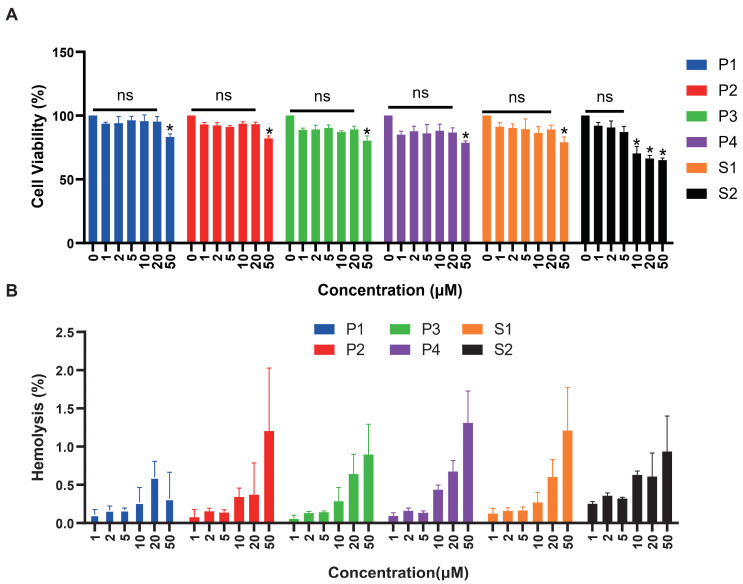
Cytotoxicity and hemolytic analysis of six peptides. (**A**) The cytotoxicity effects of antimicrobial peptides at different concentrations were tested on HEK293T cells. Peptide concentration (μM) was plotted on the x-axis, and cell viability (%) on the y-axis (mean ± SD, *n* = 5). ns: no statistical significance, *: *p* < 0.05. (**B**) Red blood cell hemolytic rates (%) measured at varying concentrations of antimicrobial peptides (μM). Peptide concentration (μM) was plotted on the x-axis, and hemolysis (%) on the y-axis (mean ± SD, *n* = 5).

We tested the cytotoxicity of six peptides with HEK293T cells (Figure 6A). The significance was compared with cell activity at 0 μM concentration. There was no cytotoxicity of P1 and P3 within the concentration range detected. Peptides P2, P4, and S1 caused significant cell death at high concentrations of 50 μM. Regarding peptide S2, cell viability did not exceed 75% at concentrations of 10, 20, and 50 μM, indicating significant cytotoxic effects at 10 μM concentration. As shown in Figure 6B, we also evaluated the hemolytic activity of all peptides, respectively. After co-incubation with red blood cell suspension for 30 min, hemolytic rates of six peptides ranged from 0.1% to 1.5%, indicating low hemolytic activity. Hemolytic rates of 1.4% and 1.3% were observed when red blood cells were incubated with 50μM of P4 peptide, 50 μM of P2 and S1, respectively.

## 3. Discussion

In our study, we identified 4490 mucous proteins in Pacific oysters, serving as a crucial barrier against pathogens in marine organisms. Actually, the epidermal mucus of fish serves as a primary source for extracting natural antimicrobial peptides [30,31]. Mucous secretions of molluscs also contain active peptides directly involved in antimicrobial function; for instance, a novel cysteine-rich antimicrobial peptide (mytimacin-AF) characterized from the mucus of the snail of *Achatina fulica* showed potent antimicrobial activity against *Staphylococcus aureus* and the fungus *Candida albicans* [32]. Nevertheless, few antimicrobial peptides were identified here in mucous proteins, which lead us to consider the possible products of peptides from the degradation or enzymatic hydrolysis. Due to variations in peptide detection sensitivity and enzymatic digestion-induced peptide diversity, conventional mass spectrometry identification methods have limitations in screening and discovering bioactive peptides in molluscs [33]. To address these issues, we integrated amino acids’ hydrophobicity scores, relative scores calculation, and comprehensive antimicrobial peptide classification networks which are particularly well suited for the analysis of large sets of sequences, including entire protein databases, enhancing our ability to predict the component structure and biological activities of these peptides.

Herein, we employed deep learning algorithms to analyze the antimicrobial peptides in Pacific oyster mucus and screened 43,000 peptides. A similar research approach was utilized by Tachapuripunya, V et al. [34], who employed k-NN and Random Forest (RF) algorithms to predict and analyze the function of trypsin peptides (1218 core peptides and 1600 variable peptides) extracted from the mucus of seven common gastropods. On this basis, we intergrated iAMPCN deep-learning framework to identify and predict the potent activity of the mucus-derived peptides in 23 functional indicators against pathogens to enhance our drug development method of peptide therapeutics. Consequently, a series of antimicrobial peptides were predicted and filtered, which offers the potential to test their activities against microbes using a bactericidal assay.

Our research on six synthetic peptides, specifically P2 and P4, has shown promising results in inhibiting the growth of both *E. coli* and *S. aureus*. This broad-spectrum antibacterial efficacy suggests potential applications in therapeutic settings where treatment of infections caused by diverse bacterial pathogens is required. This is particularly significant considering the variability in the effectiveness of existing antimicrobial peptides, such as those derived from *Crassostrea gigas* (Cg-Defs) [35]. Cg-Defs have been primarily effective against Gram-positive bacteria, with minimal inhibitory concentrations ranging from 0.01 to 6 μM. However, their efficacy diminishes against Gram-negative bacteria, with generally higher MICs exceeding 10 μM. This limited activity can be attributed to the protective outer membrane of Gram-negative bacteria that shields targets like peptidoglycan from the action of these peptides [36]. In contrast, the broad-spectrum activity observed in our peptides P2 and P4 could be due to a more versatile mechanism of action, potentially engaging multiple bacterial targets or pathways, thereby enhancing their antimicrobial reach. This versatility aligns with findings from other researchers like Louis et al. [37], who synthesized five peptides that effectively targeted various strains within the *Vibrio* genus using a novel PepTraq prediction tool combining transcriptomic differential analysis in the blood cells of the common cuttlefish *Sepia officinalis*. Similar to peptide GK28 from their study [37], which demonstrated high activity against *Vibrio* species without inducing hemolysis, our peptides P2 and P4 also showed notable anti-*V. parahaemolyticus* activity combined with safety. Therefore, our study highlights the potential for in-depth computational analysis to uncover novel antimicrobial peptides, highlighting the potential application of these peptides in future antimicrobial therapies.

The integration of machine learning techniques with experimental approaches can significantly enhance the development of antimicrobial peptides [38]. The low toxicity and safety predicted by deep learning have also been verified in actual experiments. Our research exemplified this by combining in silico analyses with proteomic data. Notably, all six peptides tested exhibited remarkably low hemolytic activity, maintaining levels below 1.5% even at high concentrations, demonstrating a favorable safety profile. This observation aligns with prior studies that searched for antimicrobial sequences in the cuttlefish (*Sepia officinalis*) database by in silico analysis of a transcriptomic database. The result showed that GR21, a peptide less than 25 amino acids in length, induced less than 10% hemolysis on human blood cells at a concentration of 200 µM [39]. Moreover, peptides P1 and P3 in our study demonstrated no cytotoxic effects on HEK293T cells across the tested concentration range, which presented a comprehensive safety assessment proving the feasibility of developing antimicrobial peptides derived from invertebrates by machine learning.

In summary, we have delineated promising research avenues concerning antimicrobial peptides (AMPs) derived from oyster mucus, with prospects for further refinement in drug design methodologies. Through proteomic analysis of *Crassostrea gigas* mucous proteins, we have compiled a valuable database of bioactive peptides ranging from eight to 50 amino acids in length. Employing the iAMPCN algorithm, we predicted biological properties across 23 categories, identifying six peptide candidates characterized by α-helical structures and favorable hydrophobic properties. Our approach integrated comprehensive amino acid sequences analysis, iAMPCN modeling for activity prediction, and in vitro assays to screen AMPs sourced from marine molluscs. Of the six candidates, four peptides demonstrated antibacterial efficacy against *E. coli* strain, with P2 and P4 showed significant effects. All peptides effectively inhibited the growth of *S. aureus*. Peptides P2 and P4 exhibited notable anti-*V. parahaemolyticus* activity, while P1 and P3 showed no cytotoxic effects of HEK293T cells within the concentration range detected. Importantly, all six peptides displayed minimal hemolytic activity, remaining below 1.5% even at high concentrations, indicating their potential suitability for clinical drug applications. Overall, the application of machine learning in antimicrobial peptide research shows promising prospects across biomedical sciences.

## 4. Materials and Methods

### 4.1. Animals Culture and Mucus Collection

Healthy Pacific oysters (*Crassostrea gigas*) were procured from Qingdao, Shandong Province, China, and acclimated in a tank of seawater with a salinity of 27‰ at approximately 20 °C for nearly 1 week prior to experimentation. During the acclimation period, the oysters were fed *Isochrysis galbana* twice daily to ensure their optimal health and readiness for the study. Following the methodology described by Pales Espinosa et al. [40], oysters were carefully opened, and their pallial organs were rinsed with sterile artificial seawater (SAS; salinity 27‰, filtered through 0.22 µm membranes) for mucus extraction. Mucus from the mantle, gills, and labial palps of each oyster was collected using sterile cotton swabs. The swabs from three oysters were pooled into a single 15 mL plastic tube containing 10 mL of ice-cold SAS and 100 µL of a protease inhibitor cocktail (Beyotime, Shanghai, China), resulting in approximately 500 µL of mucus per sample. The tubes were gently agitated at 4 °C on a rotating shaker for 2 h and the collected fluid was then centrifuged at 4 °C for 20 min at 1000× *g*. The supernatant was filtered through 0.22 µm sterile syringe filters to remove debris. Samples were maintained at 4 °C until use, which were conducted within the following hour. Three samples were obtained for further exploration.

### 4.2. Protein Extraction

The samples were mixed with five volumes of cold acetone and left to precipitate overnight. The fluid was then centrifuged at 4 °C for 15 min at 25,000× *g*. The resulting pellet was air-dried and redissolved in a solution containing 10 mM DTT (DL-Dithiothreitol). They were then sonicated in an ice bath (frequency is 50 Hz, time is 3 min) and centrifuged at 25,000× *g*, 4 °C for 15 min to collect the supernatant. DTT with a final concentration of 10 mM was added again, 56 °C water bath for 1 h. Subsequently, 55 mM IAM (Iodoacetamide) was added to the mixture, which was then incubated in the dark for 45 min. Following incubation, the mixture was centrifuged at 25,000× *g*, 4 °C for 15 min, and the supernatant was collected as the protein solution. A 25 µL aliquot of each fluid was assessed for protein concentration using the Modified BCA Protein Assay Kit (Sangon Biotech, Shanghai, China) following the manufacturer’s guidelines. The fluids were then diluted with SAS to achieve a final protein concentration of 1 mg/mL [41].

### 4.3. Proteolysis and Peptide Fractionation

First, 100 μg protein was taken and added to a 1.5 mL centrifuge tube. Trypsin was used to digest the proteins at a ratio of protein to trypsin of 20:1 at 37 °C for 4 h. After digestion, the peptide liquid was taken out for desalting and then freeze-dried for further analysis Next, the mixed 20 μg sample was taken for fractionation using the LC-20AB liquid phase system (Shimadzu, Tokyo, Japan) which was equipped with a 5 μm 4.6 × 250 mm Gemini C18 column. The dried peptide samples were redissolved in mobile phase A (5% ACN, pH 9.8) and separated at a flow rate of 1 mL/min with the following gradient: 5% mobile phase B (95% ACN, pH 9.8) for 10 min, 5% to 35% mobile phase B over 40 min, 35% to 95% mobile phase B over 1 min, 95% mobile phase B for 3 min, and 5% mobile phase B for 10 min. The elution peak was monitored at a wavelength of 214 nm and one component was collected per minute, and the samples were combined according to the chromatographic elution peak map to obtain 20 fractions, which were then freeze dried.

### 4.4. LC-MS/MS

The freeze-dried peptide samples were redissolved with mobile phase A (2% ACN, 0.1% FA) and centrifuged at 20,000× *g* for 10 min, and the supernatant was taken for injection. Separation was performed by UltiMate 3000 UHPLC (Thermo Scientific, Waltham, MA, USA). The sample was first enriched in trap column and desalted, and then entered a self-packed C18 column (75 μm internal diameter, 3 μm column size, 25 cm column length) and separated at a flow rate of 300 nL/min. The nanoliter liquid phase separation end was directly connected to the mass spectrometer. Subsequently, the peptides separated by liquid phase chromatography were ionized by a nanoESI source followed by passing to a tandem mass spectrometer Q-Exactive HF X (Thermo Fisher Scientific, San Jose, CA, USA) for DDA (Data Dependent Acquisition) mode detection. The parameters for MS analysis are as follows: ion source voltage was set to 1.9 kV, MS1 scanning range was 350~1500 *m*/*z*, resolution was set to 60,000, MS2 starting *m*/*z* was fixed at 100, and resolution was 30,000. The ion screening conditions for MS2 fragmentation were as follows: charge 2+ to 6+, and the top 20 parent ions with the peak intensity exceeding 20,000. The ion fragmentation mode was HCD, and the fragment ions were detected in Orbitrap. The dynamic exclusion time was set to 30 s. The AGC was set to MS1 3E6, MS2 1E5.

### 4.5. Protein Identification and Bioinformatics Analysis

The raw MS/MS data were converted into MGF format by thermo scientific tool Proteome Discoverer (version 2.5), and the proteins were searched using Mascot version 2.3.02 against the Uniprot database (https://www.uniprot.org/, accessed on 23 February 2023). In order to control the rate of false positive results at the protein level, 1% of protein FDR, based on the picked protein FDR strategy, was also set as the criteria for protein identification. As a result, proteins containing at least one unique set of spectra with filtration of FDR ≤ 1% were served as downstream analysis. The identified peptides were applied to GO annotation by Blast2GO (version 5.2). Pathway enrichment analysis was performed by using a search pathway tool in the KEGG Mapper platform (https://www.genome.jp/kegg/, accessed on 10 March 2023).

### 4.6. Generation and Analysis of Oyster Mucus Peptide Libraries

The generation and analysis of oyster mucus peptide libraries referenced the methodology proposed by Katia et al. [29]. For the generation of peptide sets, the initial hydrophobicity scale of amino acids was obtained by measuring the retention time of peptide in the column firstly. The amino acids “*X*” with the lowest hydrophobicity were selected as reference, and the amino acid “*Z*” with the highest hydrophobicity score was found. Next, the hydrophobicity fraction “*H*′*_A_*” for any amino acid was calculated using a specified formula, *H*′*_A_*= (*H_A_* − *H_X_*)/(*H_Z_* − *H_X_*), in the newly generated scale, ensuring that the overall hydrophobicity scores of the peptide were determined only by the amino acids with higher hydrophobicity. Due to the enormous size of the mucosal protein-derived peptide dataset from large-scale proteomic data, fully retrieving it exceeds current computational capabilities. Consequently, the peptides used in this study represent a random sample of the overall data. Given the importance of function and stability in antimicrobial peptides (AMPs) ranging from eight to 50 amino acids, 1000 peptides of each length were selected for a diverse analysis. This approach led to the examination of 43,000 polypeptides, ensuring a broad representation while managing computational limits.

Based on the amino acids scores, the relative score (RS) for amino acids were determined using residues properties as parameters and the correlation with peptide sequence length was established through absolute score. Specifically, “*C*” is the net charge of the peptide, “*H*” is the sum of the hydrophobicity scores of all the peptide residues, “*L*” is the number of peptide residues, and “*MaxScore*” is the highest *CmHn* value; the formula is as follows:

*Relative Score* (*RS*) = (*CmHn*)/*MaxScore*

*Absolute Score* (*AS*) = *RS* × *L*

Antimicrobial scores for amino acids were computed, followed by an evaluation of the antimicrobial potential of the peptides.

### 4.7. iAMPCN Model Prediction on Polypeptides Functional Activity

A novel deep-learning framework known as iAMPCN (identification of AMPs and their functional activities based on Convolutional Neural Networks) was introduced by Jing et al. [28]. The source code for iAMPCN can be found at (https://github.com/joy50706/iAMPCN/tree/master, accessed on 8 April 2024). This framework aims to enhance the predictive accuracy of AMPs and their functional activities. Specifically, iAMPCN utilizes one-hot coding to convert the amino acid sequences of antimicrobial peptides into numerical representations that can be processed by computers. A CNN (Convolutional Neural Network) model is constructed using convolutional and pooling layers to extract features from antimicrobial peptide sequences. The model is capable of predicting the functional activities of antimicrobial peptides and optimizing the results. Therefore, the 23 functional indicators, including antibacterial, antifungal, and antiviral properties were predicted. Subsequently, polypeptides with the RS top four that concentrated in broad antibacterial activity, low toxicity, and low hemolytic activity were selected and named as P1–P4; the two highest RS of 43,000 peptides were named as S1 and S2 for subsequent analysis. These peptides were selected as representative of the reliability of the analytical method and these properties were the optimal parameters for screening valuable peptides.

### 4.8. Polypeptides Physicochemical Properties Prediction

The three-dimensional structure of peptides set size from eight to 50 amino acid residues was predicted through the PEP-FOLD (https://bioserv.rpbs.univ-paris-diderot.fr/services/PEP-FOLD3/, accessed on 21 June 2024) website [42] and subjected to analysis by PyMOL software (version 2.60). The physicochemical properties of peptides were accomplished by Heliquest (https://heliquest.ipmc.cnrs.fr/, accessed on 21 June 2024), with regard to hydrophobicity, hydrophobic moment, and net charge [43].

### 4.9. Antimicrobial Activity Assay

Candidate peptides were assessed for antimicrobial activity against *Escherichia coli* DH5α, *Vibrio parahaemolyticus* E151, and *Staphylococcus aureus* ATCC 29213 using the method of Mao et al. [44] with some modifications. Minimum inhibitory concentrations (MICs) were determined using broth microdilution in LB medium, starting with an inoculum of 1 × 10^5^ cells in untreated polystyrene microtiter plates. LB broth without cells served as the blank control. Peptides were tested at concentrations of 0, 1, 2, 5, 10, 20, and 50 μM in LB broth with cells. Following a 12 h incubation at 37 °C, OD at 600 nm readings were taken in a microplate reader to assess bacterial growth inhibition. Data were presented as a heat map depicting antimicrobial efficacy against the three bacterial strains. Each experiment was performed in triplicate for reliability, and heat map values represent the mean OD (600) of replicates after normalization against the blank control.

### 4.10. Cytotoxicity Assay

HEK293T cells were cultured in DMEM (10% FBS, 1% penicillin–streptomycin solution) in a 37 °C, 5% CO_2_ cell culture incubator until reaching the logarithmic growth phasein. As previously described [45], cells were seeded into a 96-well plate at 100 μL per well and pre-incubated for 48 h. After removing the medium, 10 μL of antimicrobial peptide solutions (1, 2, 5, 10, 20, and 50 μM) were added to each well with five replicate wells per concentration. The HEK293T cells cultured with DMEM (0 μM peptide) constituted the untreated group and plates with no cells were as blank group. Plates were then cultured for 24 h. Subsequently, 10 μL of CCK-8 solution was added to each well and incubated for 3 h under the same conditions using the Cell Counting Kit-8 (CCK-8, K1018, APE×BIO, Technology LLC, Houston, TX, USA). Absorbance at 450 nm was measured using a microplate reader. Cell viability was calculated using the following formula: Cell viability (%) = [Treated group OD (450) − Blank group OD (450)]/[Untreated group OD (450) − Blank group OD (450)] × 100%.

### 4.11. Hemolysis Assay

We employed the method described by Sæbø et al. [46]. Blood from fiber sheep was centrifuged at 1000 rpm for 10 min to isolate red blood cells (RBCs). The supernatant was removed, and RBCs were washed with PBS until the supernatant cleared. RBCs were resuspended in PBS at a 10-fold volume ratio to create a uniform RBC suspension. Antimicrobial peptides were diluted in PBS to concentrations of 1, 2, 5, 10, 20, and 50 μM using serial dilution. Subsequently, 10 μL of each peptide dilution was added to separate wells of a 96-well plate for experimental groups. Negative controls received 10 μL PBS, and positive controls contained 10% Triton X-100. Each well received 1 × 10^5^ RBCs, with five replicate wells per concentration. The plate was then incubated at 37 °C with 5% CO_2_ for 30 min. Following incubation, the plate was centrifuged at 1500 rpm for 10 min, and the supernatant was transferred to a new 96-well plate. Optical density at 450 nm was measured using a spectrophotometer. Hemolysis percentage was calculated using the following formula: Hemolysis % = [Experimental group OD (450) − Negative control OD (450)]/[Positive control OD (450) − Negative control OD (450)] × 100%.

### 4.12. Statistical Analysis

Statistical analysis was performed using GraphPad Prism (version 9.5.0) and was conducted using one-way analysis of variance (ANOVA) followed by Tukey’s test. Statistical significance values are indicated as *: *p* < 0.05.

## Figures and Tables

**Table 1:** Summary of protein identification results.

Sample Name	Total Spectra	Spectra	Unique Spectra	Peptide	Unique Peptide	Protein
*Crassostrea_gigas*	388,065	39,974	29,987	18,642	15,113	4490

**Table 2:** Sequence information, amino acid score, and functional prediction results of six peptides screened by machine learning.

	Sequence	Score	AMP	Antibacterial	Anti-Gram-Positive	Anti-Gram-Negative	Antifungal	Antiviral	Anti-Mammalian-Cells	Hemolytic	Cytotoxic	Endotoxin
**P** **1**	FAKLLLRLPALR	0.602235	Yes	Yes	Yes	Yes	Yes	No	No	No	No	No
**P** **2**	WKQIKFYVYNRILKKRK	0.565195	Yes	Yes	Yes	Yes	Yes	No	No	No	No	No
**P** **3**	KVWQRVKVKML	0.564499	Yes	Yes	Yes	Yes	Yes	No	No	No	No	No
**P4**	RRRAKKMRAIYLIMNRFKKYKI	0.562785	Yes	Yes	Yes	Yes	Yes	No	No	No	No	No
**S1**	LLFRRRLL	0.947085	Yes	Yes	Yes	Yes	No	No	Yes	No	No	Yes
**S2**	LLRRKFFF	0.927233	Yes	Yes	Yes	Yes	No	No	Yes	No	No	Yes

## Data Availability

The original data presented in the study are included in the Supplementary Material; further inquiries can be directed to the corresponding author.

## Supplementary Materials

The following supporting information can be downloaded at: https://www.mdpi.com/article/10.3390/md22090385/s1, We have provided two Excel files as supplementary data. File S1 contains 8–50 amino acids peptide dataset. File S2 includes machine learning prediction results. These files support the findings presented in our manuscript and can be accessed for detailed data analysis.
